# Supplementary material for: Anemia in female patients with myasthenia gravis
Source: PLoS One. 2022 Sep 6;17(9):e0273720. doi: 10.1371/journal.pone.0273720 (PMC9447896; doi:10.1371/journal.pone.0273720)
Supplement: S1 Table — * One patient has Grave’s disease and neuromyelitis optica spectrum disorders, and one patient has Grave’s disease and stiff-person syndrome, one patient has Systemic lupus erythematosus and Sjögren’s syndrome. ** One patient has Hashimoto’s disease and type 1 diabetes mellitus. (DOCX) [file pone.0273720.s002.docx]

**S1 Table.**

|  | anemia(N=85)* | non-anemia(N=130)** |
| --- | --- | --- |
| Hashimoto’s disease | 4 | 15 |
| Grave’s disease | 2 | 7 |
| Antithyroid antibody positive, but no symptom | 1 | 2 |
| Type 1 diabetes mellitus | 2 | 3 |
| Stiff-person syndrome | 1 | 0 |
| Rheumatoid arthritis | 2 | 4 |
| Systemic lupus erythematosus | 1 | 0 |
| Sjögren’s syndrome | 1 | 1 |
| Systemic sclerosis | 0 | 1 |
| Anti-phospholipid antibody syndrome | 0 | 1 |
| Myositis | 3 | 0 |
| Sarcoidosis | 1 | 0 |
| Ulcerative colitis | 1 | 0 |
| Crohn's disease | 1 | 0 |
| Asthma | 0 | 2 |
| Neuromyelitis optica spectrum disorders | 1 | 0 |
| Psoriasis | 0 | 1 |

* One patient has Grave’s disease and neuromyelitis optica spectrum disorders, and one patient has Grave’s disease and stiff-person syndrome, one patient has Systemic lupus erythematosus and Sjögren’s syndrome.

** One patient has Hashimoto’s disease and type 1 diabetes mellitus
