# Supplementary material for: Anemia in female patients with myasthenia gravis
Source: PLoS One. 2022 Sep 6;17(9):e0273720. doi: 10.1371/journal.pone.0273720 (PMC9447896; doi:10.1371/journal.pone.0273720)
Supplement: S2 Table — CNIs: calcineurin inhibitors, QMG score: quantitative myasthenia gravis score; SD: standard deviation. *: p < 0.05, **: p < 0.01. (DOCX) [file pone.0273720.s003.docx]

**S2 Table.** Comparison between female MG patients with and without moderate or severe anemia at latest hemoglobin level

|  | **Current anemia**  **(N=21)** | **Current non-anemia**  **(N=194)** | ***p*-value** |
| --- | --- | --- | --- |
| Prednisolone, % | 85.7 | 66.5 | 0.0720 |
| CNIs, % | 71.4 | 43.3 | 0.014 * |
| Worst QMG score, mean ±SD | 16.1 ±6.4 | 14.0 ±6.1 | 0.1331 |
| MG-ADL, mean ±SD | 7.4 ±5.1 | 5.4 ±4.2 | 0.0695 |
| MG composite, mean ±SD | 10.5 ±7.8 | 7.0 ±6.6 | 0.0207 * |
| MG-QOL15r, mean ±SD | 15.1 ±7.6 | 10.3 ±7.4 | 0.0041 ** |

CNIs: calcineurin inhibitors, QMG score: quantitative myasthenia gravis score; SD: standard deviation.

*: *p* < 0.05, **: *p* < 0.01
