# Supplementary material for: Anemia in female patients with myasthenia gravis
Source: PLoS One. 2022 Sep 6;17(9):e0273720. doi: 10.1371/journal.pone.0273720 (PMC9447896; doi:10.1371/journal.pone.0273720)
Supplement: Attachment — Submitted filename: Response to reviewers_0620.docx [file pone.0273720.s004.docx]

June 24, 2022

RE: Revised PONE-D-22-12752

Anemia in female patients with myasthenia gravis

Dear Dr. Jong-Ling Fuh

Thank you very much for your letter of May 28, 2022, regarding our manuscript entitled, " Anemia in female patients with myasthenia gravis". We thank the reviewers for their comments; our responses and the changes made (or reasons for not making changes) are summarised below.

| **Reviewer’s comment** | **Response** | **Changes made** |
| --- | --- | --- |
| ***Reviewer #1*** | | |
| Iron deficiency anemia (IDA) was the most common cause of anemia in this cohort (49/85=58%) and controlled by iron supplementation. The underlying causes of IDA should be provided and the response to iron supplementation should be analyzed also. | We appreciate the kindly comments.  Among 49 MG patients with iron deficiency anemia, 35 (71%) had hemoglobin levels within the normal range at the time of the latest hemoglobin count by iron supplementation. | We had added the following  sentence on Page 8: “Among 49 MG patients with iron deficiency anemia, 35 (71%) had hemoglobin levels within the normal range at the time of the latest hemoglobin count by iron supplementation.”. |
| The second most common cause of anemia was presumed to be drug-related in 32% of 85 MG patients based on lack of possible underlying disease contributory to anemia. Was this subgroup of anemic patient more resistant to anemia treatment? Subgroup analysis of anemia with all MG outcome measures might provide more clues for the true impact of MG drugs on QoL. | There was no clear difference in the latest hemoglobin levels between the anemia group with unknown cause and the anemia group with cause (12.5±1.7, 12.2±1.9, p=0.46 ). | We had added the following  sentence on Page 8: “There was no clear difference in the latest hemoglobin levels between the anemia group with unknown cause and the anemia group with cause (12.5±1.7, 12.2±1.9, p=0.46 ).”. |
| The total number of MG cohort seems to be inconsistent. 383 patients in page 4 (Patients and methods) but 385 patients in page 5 (Results). | Thank you for your kind remarks. 388 is the exact number of people as shown in the study flow. | The description on Page 4 has been corrected. |
| Were the quantitative assessments including QMG, MG-ADL, and MG-QoL performed on routine basis or at the last outpatient visit? | We appreciate the important suggestion. The QMG scores were evaluated in the worst condition during the entire courses. In contrast, the MG-ADL, MG composite, and the revised scale of the 15-item MG-QOL were recorded in the last outpatient visit. Due to COVID-19 pandemic, we could not easily perform pulmonary function tests. Thus, we utilized MG composite instead of QMG score. | We had added the following sentences on Page 4. “The quantitative (QMG) scores were evaluated in the worst condition during the entire courses. In contrast, the MG-ADL, MG composite, and the revised scale of the 15-item MG-QOL were recorded in the last outpatient visit. Due to COVID-19 pandemic, we could not easily perform pulmonary function tests. Thus, we utilized MG composite instead of QMG score.” |
| ***Reviewer #2*** | | |
| The purpose of this study was to investigate factors associated with anemia and the negative impact of anemia in female MG patients. The authors investigated factors related to MG and anemia in 215 female patients with MG. According to their results 40% of the female MG patients had moderate or severe anemia after the diagnosis of MG, female MG patients with anemia presented with severe muscle weakness, which resulted in their receiving more intensive immunotherapy, 32% of the patients had no known cause of anemia, and hemoglobin levels were associated with the revised scale of the 15-item MG-QOL. They conclude that long-term immunotherapy for MG may cause various side effects, including anemia in female MG patients. It is a very interesting study on MG, since so far prevalence and causes of anemia and impact of anemia on the QOL of MG patients have not been elucidated. Important study for the clinicians, as the negative impact of anemia in MG patients may be prevented. | Thank you for your favorable comments! |  |
| ***Reviewer #3*** |  |  |
| The authors should have considered several confounding factors when analyzing the association between anemia and MG severity and quality of life. In particular, since long-term immunosuppressive treatment is related to both anemina and mg severity, it is necessary to adjust the effect using methods such as stratification or multivariate regression. | We think this suggestion is important. Bulbar involvement, QMG score, long-term immunosuppressive treatment with prednisone and calcineurin inhibitors were associated with anemia in MG patients. These factors were entered into multiple linear regression analysis for  determination of independent background factors  associated with lowest hemoglobin value. Only the QMG score suggested multiple correlations (r=-0.72, *p*=0.018), but the other items were not statistically significant. When compared with and without moderate to severe anemia in terms of current hemoglobin levels, CNIs were significantly higher in the group with current anemia.MG composite and MG-QOL15r were significantly higher in the anemic group.(We added Supplementary Table 2) | We had added the following sentences on Page 12. “Another limitation is that anemia in female MG patients may be due to long-term use of immunosuppressive drugs, but the use of immunosuppressive drugs is also a confounding factor with the severity of MG. When the groups were divided by the presence or absence of anemia in the latest hemoglobin level, the worst QMG score ever was not statistically significant. On the other hand, the use of calcineurin inhibitors was statistically significantly higher in the anemic group (S2 Table).” |

We believe that these suggestions have been helpful in improving the manuscript and look forward to its publication in PLOS One. If there are any questions regarding the manuscript, please do not hesitate to contact me.

Sincerely yours,

Shigeaki Suzuki, MD, PhD
