# Supplementary material for: Anemia in female patients with myasthenia gravis
Source: PLoS One. 2022 Sep 6;17(9):e0273720. doi: 10.1371/journal.pone.0273720 (PMC9447896; doi:10.1371/journal.pone.0273720)
Supplement: Attachment — Submitted filename: Response to Reviewers.docx [file pone.0273720.s005.docx]

August 10, 2022

RE: Revised PONE-D-22-12752

Anemia in female patients with myasthenia gravis

Dear Dr. Jong-Ling Fuh

Thank you very much for your letter of August 2, 2022, regarding our manuscript entitled, " Anemia in female patients with myasthenia gravis". We thank the reviewers for their comments; our responses and the changes made (or reasons for not making changes) are summarised below.

| **Editor’s comment** | **Response** | **Changes made** |
| --- | --- | --- |
| Please revise the last paragraph (Discussion section, conclusion part) with key messages that the article intends to deliver as the reviewer suggested. | We apologize for not writing in accordance with the PLOS ONE method. I have added a concluding paragraph at the end. | We had removed the last sentence of “Discussion” and added the following  sentence on Page 13: “Conclusions With modern immunosuppressive, symptomatic, and supportive treatments, the prognosis for MG patients is generally favorable. However, long-term drug treatment is usually necessary for most MG patients. In addition to various adverse events of corticosteroids and non-steroidal immunosuppressants, female MG patients suffer from anemia. Anemia which is suspected to be related to long-term immunosuppressive drugs, causes the impairment of patients’ QOL. Monitoring hemoglobin levels is necessary for best management of female MG patients.”. |

We believe that these suggestions have been helpful in improving the manuscript and look forward to its publication in PLOS One. If there are any questions regarding the manuscript, please do not hesitate to contact me.

Sincerely yours,

Shigeaki Suzuki, MD, PhD
